# Supplementary material for: The Validity of the Original and the Saudi-Modified Screening Tools for the Assessment of Malnutrition in Pediatrics: A Cross-Sectional Study
Source: Diagnostics (Basel). 2024 Oct 10;14(20):2256. doi: 10.3390/diagnostics14202256 (PMC11505708; doi:10.3390/diagnostics14202256)
Supplement: Supplementary file 1 [file diagnostics-14-02256-s001.zip › diagnostics-3248878-supplementary.pdf]

Supplementary Material S1.

The original screening tool for the assessment of malnutrition in pediatrics:

Step 1 – Diagnosis	
Does the child have a diagnosis that has any nutritional implications?	Score
Definitely	3
Possibly	2
No	0

Step 2 – Nutritional intake	
What is the child's nutritional intake?	Score
None	3
Recently decreased/poor	2
No change/good	0

Step 3 – Weight and height	
Use a growth chart or the centile quick reference tables to determine the child's measurements	Score
> 3 centile spaces/ ≥ 3 columns apart (or weight < 2nd centile)	3
> 2 centile spaces/ = 2 columns apart	1
0 to 1 centile spaces/ columns apart	0

Step 4 – Overall risk of malnutrition	
Add the scores from steps 1–3 together to calculate the overall risk of malnutrition	Score
High risk	≥ 4
Medium risk	2–3
Low risk	0–1

Step 5 – Care plan		
Develop a care plan based on the child's overall risk of malnutrition		
High risk	Medium risk	Low risk
▪ Take action<br>▪ Refer to a Dietitian, nutritional support team or consultant<br>▪ Monitor as per care plan	▪ Monitor nutritional intake for 3 days<br>▪ Repeat STAMP screening after 3 days<br>▪ Amend care plan as required	▪ Continue routine clinical care<br>▪ Repeat STAMP screening weekly while child is an in-patient<br>▪ Amend care plan as required

Definite nutritional implications	Possible nutritional implications	No nutritional implications
▪ Bowel failure, intractable diarrhoea<br>▪ Burns and major trauma<br>▪ Crohn's disease<br>▪ Cystic fibrosis<br>▪ Dysphagia<br>▪ Liver disease<br>▪ Major surgery<br>▪ Multiple food allergies/intolerances<br>▪ Oncology on active treatment<br>▪ Renal disease/failure<br>▪ Inborn errors of metabolism	▪ Behavioural eating problems<br>▪ Cardiology<br>▪ Cerebral palsy<br>▪ Cleft lip and palate<br>▪ Coeliac disease<br>▪ Diabetes<br>▪ Gastro-oesophageal reflux<br>▪ Minor surgery<br>▪ Neuromuscular conditions<br>▪ Psychiatric disorders<br>▪ Respiratory syncytial virus (RSV)<br>▪ Single food allergy/intolerance	▪ Day case surgery<br>▪ Investigations

INFANT WEIGHT AND HEIGHT CENTILE TABLES

STAMP
SCREENING TOOL FOR THE ASSESSMENT OF MALNUTRITION IN PAEDIATRICS

Infant weight centile tables – boys

Weight centiles (kg)									
**Age**	**0.4th**	**2nd**	**9th**	**25th**	**50th**	**75th**	**91st**	**98th**	**99.6th**
**Birth**	2.17	2.5	2.83	3.16	3.5	3.84	4.17	4.5	4.84
**2 months**	3.95	4.3	4.7	5.12	5.56	6.05	6.55	7.2	7.65
**4 months**	5.15	5.5	6	6.5	7	7.55	8.15	8.75	9.4
**6 months**	5.9	6.35	6.85	7.4	7.94	8.55	9.2	9.85	10.6
**8 months**	6.45	6.9	7.45	8	8.6	9.26	9.95	10.65	11.45
**10 months**	6.85	7.3	7.9	8.5	9.2	9.8	10.6	11.45	12.2
**12 months**	7.2	7.7	8.3	8.95	9.65	10.4	11.2	11.95	12.9
**14 months**	7.5	8.1	8.7	9.37	10.1	10.9	11.7	12.6	13.5
**16 months**	7.85	8.4	9.1	9.75	10.5	11.4	12.2	13.2	14.05
**18 months**	8.1	8.7	9.4	10.1	10.95	11.8	12.7	13.7	14.7
**20 months**	8.4	9	9.75	10.5	11.35	12.3	13.2	14.15	15.25
**22 months**	8.7	9.4	10.1	10.9	11.75	12.7	13.7	14.7	15.9
**24 months**	9	9.7	10.4	11.3	12.1	13.1	14.2	15.3	16.4

Infant height centile tables – boys

Height centiles (cm)									
**Age**	**0.4th**	**2nd**	**9th**	**25th**	**50th**	**75th**	**91st**	**98th**	**99.6th**
**Birth**	44.8	46.1	47.9	49.5	51	52.5	54.1	55.9	57.1
**2 months**	53.1	54.5	55.8	57.1	58.4	59.8	61.1	62.5	63.7
**4 months**	58.3	59.5	61.1	62.5	63.9	65.3	66.7	68	69.4
**6 months**	62	63	64.8	66.2	67.6	69.1	70.5	72	73.3
**8 months**	64.7	66	67.6	69.1	70.6	72.1	73.5	75	76.5
**10 months**	67.2	68.5	70.2	71.7	73.3	74.8	76.4	77.8	79.3
**12 months**	69.5	70.8	72.5	74.1	75.8	77.3	78.9	80.5	82
**14 months**	71.5	73	74.8	76.4	78.1	79.7	81.4	83	84.6
**16 months**	73.4	75	76.8	78.5	80.2	82	83.6	85.5	87
**18 months**	75	76.8	78.6	80.4	82.2	84.1	85.8	87.8	89.5
**20 months**	76.8	78.5	80.5	82.3	84.2	86.1	87.9	89.8	91.6
**22 months**	78.3	80	82.1	84.1	86	88	89.9	92	93.8
**24 months**	79	81	83	85.1	87.1	89.2	91	93.5	95.3

* Developed from UK-WHO growth charts. Growth charts may also be used to assign a score for step 3 of STAMP

© 2008 Central Manchester and Manchester Children's University Hospitals NHS Trust
Supported by an educational grant from Abbott.
Date Reviewed: July 2022 UK-N/A-2200464

INFANT WEIGHT AND HEIGHT CENTILE TABLES

STAMP

SCREENING TOOL FOR THE ASSESSMENT OF MALNUTRITION IN PAEDIATRICS

Infant weight centile tables – girls

Weight centiles (kg)									
Age	0.4th	2nd	9th	25th	50th	75th	91st	98th	99.6th
Birth	2.1	2.4	2.7	3.04	3.36	3.68	4	4.3	4.6
2 months	3.6	3.9	4.3	4.7	5.12	5.6	6.1	6.6	7.2
4 months	4.6	5	5.45	5.9	6.45	6.98	7.6	8.2	8.9
6 months	5.3	5.7	6.2	6.73	7.3	7.94	8.6	9.3	10.1
8 months	5.8	6.2	6.75	7.32	7.95	8.64	9.4	10.2	11
10 months	6.2	6.6	7.2	7.8	8.47	9.2	10	11	11.8
12 months	6.5	7	7.6	8.25	8.95	9.72	10.6	11.5	12.5
14 months	6.85	7.3	8	8.65	9.4	10.2	11.1	12.1	13.2
16 months	7.2	7.9	8.35	9	9.8	10.7	11.6	12.7	13.8
18 months	7.5	8	8.7	9.4	10.2	11.1	12.1	13.2	14.4
20 months	7.8	8.3	9	9.8	10.65	11.6	12.6	13.8	15
22 months	8.1	8.7	9.4	10.2	11.1	12	13.1	14.3	15.6
24 months	8.35	9	9.75	10.6	11.5	12.5	13.6	14.9	16.2

Infant height centile tables – girls

Height centiles (cm)									
Age	0.4th	2nd	9th	25th	50th	75th	91st	98th	99.6th
Birth	44.7	46	47.3	48.6	50	51.3	72.7	54	55.3
2 months	51.6	53	54.3	55.7	57	58.5	59.8	61.1	62.5
4 months	56.3	57.5	59.2	60.6	62.1	63.5	65	66.5	67.9
6 months	59.7	61	62.7	64.2	65.7	67.3	68.8	70.3	71.7
8 months	62.5	64	65.6	67.1	68.8	70.3	71.9	73.5	75
10 months	65	66.5	68.2	69.8	71.5	73.2	74.8	76.4	78
12 months	67.1	69.5	70.5	72.3	74	75.7	77.4	79.2	80.8
14 months	69.3	71	72.8	74.6	76.4	78.2	80	81.7	83.5
16 months	71.3	73	74.8	76.7	78.6	80.5	82.4	84.2	86
18 months	73	75	76.8	78.7	80.7	82.7	84.6	87	88.5
20 months	74.8	76.5	78.6	80.7	82.7	84.7	86.7	88.7	90.6
22 months	76.3	78.3	80.5	82.5	84.6	86.7	88.8	91	92.8
24 months	77.2	78.9	81.3	83.5	85.7	87.9	90	92.5	94.3

* Developed from UK-WHO growth charts. Growth charts may also be used to assign a score for step 3 of STAMP

© 2008 Central Manchester and Manchester Children's University Hospitals NHS Trust
Supported by an educational grant from Abbott.
Date Reviewed: July 2022 UK-N/A-2200464

CHILD WEIGHT AND HEIGHT CENTILE TABLES

STAMP
SCREENING TOOL FOR THE ASSESSMENT OF MALNUTRITION IN PAEDIATRICS

Child weight centile tables – boys

Weight centiles (kg)									
Age	0.4th	2nd	9th	25th	50th	75th	91st	98th	99.6th
2 years	9	9.7	10.4	11.25	12.2	13.14	14.2	15.3	16.4
2.5 years	9.75	10.5	11.4	12.3	13.3	14.4	15.6	16.9	18.1
3 years	10.4	11.2	12.2	13.2	14.3	15.5	16.9	18.3	19.9
3.5 years	11.1	12	13	14.1	15.3	16.7	18.1	19.9	21.4
4 years	12.4	13.3	14.2	5.3	16.5	17.9	19.4	21.1	23
4.5 years	13.1	14	15.1	16.2	17.6	19	20.7	23	24.6
5 years	13.9	14.9	16	17.2	18.6	20.2	22	24.1	26.5
5.5 years	14.6	15.5	16.8	18.1	19.7	21.5	23.5	26	29
6 years	15.4	16.5	17.7	19.1	20.8	22.7	25	27.8	30.7
6.5 years	16.1	17	18.5	20.1	21.9	24	26.5	29.5	33
7 years	17	18	19.5	21.1	23.1	25.4	28	31.9	35.5
7.5 years	17.6	19	20.5	22.2	24.3	27	30	34	38.7
8 years	18.5	19.5	21.5	23.3	25.6	28.4	32	36.5	42
8.5 years	19.4	20.5	22.5	24.5	27.0	30	34	39	45.8
9 years	20.2	21.8	23.5	25.7	28.4	31.8	36	42	49.5
9.5 years	21	22.5	24.6	27	29.8	33.5	38.3	44.5	53
10 years	22	23.5	25.8	28.3	31.4	35.3	40.5	47	57
10.5 years	23	24.8	27	29.7	33.0	37.2	42.8	50	60.5
11 years	24	26	28.2	31	34.6	39	45	53	64
11.5 years	24.8	27	29.4	32.5	36.3	41	47.5	55.5	67
12 years	25.8	28	30.8	34	38.1	43.2	50	58	70
12.5 years	27	29.5	32.5	36	40.4	46	53	61.5	73
13 years	28	31	34.3	38.1	43.0	49	56	65	76
13.5 years	29.8	33	36.5	40.8	46.0	52.3	60	69	80
14 years	31.5	35	39	43.6	49.2	56	63.5	73	84.5
14.5 years	33.5	37	41.5	46.5	52.3	59.5	67.5	77	88.5
15 years	35.5	39.5	44	49.1	55.4	62.7	71	81	92.5
15.5 years	38	42	46.7	52	58.1	65.5	74	84	95.5
16 years	40.5	44.7	49	54.5	60.6	68	76	86	97
16.5 years	43	47	51.5	56.5	62.6	69.5	77.7	87	98
17 years	45	49	53.2	58.3	64.3	71	79	88.1	99
17.5 years	46.5	50	54.7	60	65.7	72.5	80	89	100
18 years	48	52	56	61	66.7	73.5	81	90	101

Child height centile tables – boys

Height centiles (cm)									
Age	0.4th	2nd	9th	25th	50th	75th	91st	98th	99.6th
2 years	79	81	83	85.1	87.1	89.2	91.2	93.5	95.3
2.5 years	83	85	87.4	89.6	91.9	94.2	96.5	99	101
3 years	86.1	88.5	91	93.6	96.1	98.6	101	103.5	106
3.5 years	89.2	92	94.5	97.2	99.9	102.5	105.1	108	110.5
4 years	91.5	95.5	97	99.7	102.5	105.2	108	111	113.5
4.5 years	94.5	97.5	100.3	103.1	106.0	108.9	111.8	115	117.5
5 years	97.5	100.5	103.5	106.5	109.6	112.5	115.7	119	121.8
5.5 years	100	103	106	109.2	112.4	115.5	118.5	122	124.8
6 years	103	106	109.5	112.6	115.9	119.2	122.5	126	129
6.5 years	105.5	109	112	115.5	118.9	122.3	125.5	129.3	132.2
7 years	108	113	115	118.5	121.9	125.4	129	132.5	135.8
7.5 years	111	114	118	121.3	124.9	128.5	132	136	139.5
8 years	113.5	117	120.5	124	127.9	131.5	135	139	142.5
8.5 years	116	119	123	127	130.6	134.5	138.2	142	145.5
9 years	118	122	125.5	129.4	133.3	137.2	141	145	149
9.5 years	120	124	128	131.8	135.8	140	144	148	152
10 years	122	126	130	134.3	138.4	142.5	146.8	151	155
10.5 years	124	128	132.5	136.7	141.0	145.3	149.5	154	158
11 years	126	130	134.5	139	143.4	148	152.5	157	161
11.5 years	127.5	132	136.5	141	145.8	150.5	155	160	164
12 years	129.5	134	139	143.5	148.4	153	158	163	167.5
12.5 years	131.5	136.5	141.5	146.5	151.4	156.5	161.5	166.5	171.5
13 years	134	139	144.5	149.5	154.8	160	165	170.5	175.5
13.5 years	137	142.5	147.5	153	158.6	164	169.5	175	180
14 years	140	146	151	156.7	162.4	168	173.5	179	184.5
14.5 years	144	149.5	155	160.2	165.9	171.5	177	182.5	188
15 years	147.5	153	158	163.5	168.9	174.5	180	185.5	190.5
15.5 years	150.1	156	161	166	171.4	176.7	182	187.5	192.5
16 years	153	158	163	168.3	173.4	178.5	183.5	189	194
16.5 years	155	159	165	169.8	174.8	179.7	184.6	189.3	194.2
17 years	156.7	161	166.3	171	175.9	180.7	185.5	190.2	195
17.5 years	157.5	162	167	171.8	176.6	181.5	186	190.6	195.2
18 years	158.5	163	167.5	172.4	177	181.8	186.5	191	195.5

* Developed from UK-WHO growth charts. Growth charts may also be used to assign a score for step 3 of STAMP

© 2008 Central Manchester and Manchester Children's University Hospitals NHS Trust
Supported by an educational grant from Abbott.
Date Reviewed: July 2022 UK-N/A-2200464

CHILD WEIGHT AND HEIGHT CENTILE TABLES

STAMP
SCREENING TOOL FOR THE ASSESSMENT OF MALNUTRITION IN PAEDIATRICS

Child weight centile tables – girls

Weight centiles (kg)									
Age	0.4th	2nd	9th	25th	50th	75th	91st	98th	99.6th
2 years	8.3	9	9.8	10.6	11.5	12.5	13.5	14.9	16.1
2.5 years	9.1	10	10.8	11.7	12.8	13.9	15	16.4	18
3 years	10	10.8	11.75	12.7	13.9	15	16.5	18	20
3.5 years	10.7	11.6	12.6	13.8	15.0	16.2	18	19.8	21.9
4 years	11.2	12.2	13.3	14.5	16.0	17.6	19.4	21.5	23.9
4.5 years	12.7	13.7	14.7	15.8	17.2	18.9	20.8	22.9	25.3
5 years	13.2	14.2	15.5	16.9	18.3	20	22	24.5	27.2
5.5 years	14	15	16.2	17.7	19.4	21.3	23.5	26	29.5
6 years	14.5	15.8	17	18.7	20.5	22.5	25	28	32
6.5 years	15.4	16.5	18	19.7	21.7	24	26.8	30	34
7 years	15.8	17.5	19	20.8	23.0	25.5	28.5	32.5	37
7.5 years	17	18.5	20	22	24.4	27.3	30.5	35	40.5
8 years	18	19	21	23.3	25.9	29	33	37.7	44
8.5 years	18.5	20	22.2	24.5	27.4	30.8	35	40	47.5
9 years	19.5	21.5	23.5	26	28.9	32.5	37	43	51
9.5 years	20.5	22.5	24.5	27.2	30.6	34.5	39.5	46	55
10 years	21.5	23.5	26	28.8	32.3	36.8	42	49	59
10.5 years	22.2	24.5	27	30.2	34.1	39	45	52	62
11 years	23	25.5	28.5	32	36.0	41	47.2	55	66
11.5 years	24.2	27	30	33.5	38.1	43.5	50	58	69
12 years	25.5	28.5	31.8	35.7	40.3	46	52.5	61	71
12.5 years	27.2	30	34	38	42.8	48.4	55	63	73
13 years	29.2	32.5	36	40.3	45.4	51	58	65.5	75
13.5 years	31.2	34	38.5	43	47.9	53.5	60	68	77
14 years	33.3	36.5	40.5	45	50.1	56	62.5	70	79
14.5 years	35	38.5	42.5	47	51.9	57.5	64	72	81
15 years	37	40	44	48.4	53.4	59	66	73.5	82
15.5 years	38	41.5	45	49.5	54.6	60.3	67	74.5	84
16 years	39	42.5	46	50.5	55.5	61.5	68	76	85
16.5 years	39.8	43	47	51.3	56.2	62	68.8	76.5	86
17 years	40.4	43.6	47.3	51.8	56.9	62.6	69.3	77	87
17.5 years	40.5	44	47.9	52.2	57.2	63	70	78	87
18 years	40.9	44	48	52.3	57.5	63.5	70.5	78	88

Child height centile tables – girls

Height centiles (cm)									
Age	0.4th	2nd	9th	25th	50th	75th	91st	98th	99.6th
2 years	77.2	79	81.4	83.5	85.7	87.9	90	92.5	94.4
2.5 years	81.4	83.7	86	88.3	90.7	93.1	95.5	98	100
3 years	85	87.5	90	92.5	95.0	97.6	100.2	103	105
3.5 years	88.3	91	93.5	96.3	99.0	101.8	104.5	107.5	110
4 years	91	93	96.1	98.8	101.5	104.3	107	110	112.4
4.5 years	93.7	96.5	99.5	102.3	105.2	108	110.8	113.5	116.5
5 years	97	100	103	106	108.9	112	115	118	120.5
5.5 years	100	103	106	109	112.2	115.4	118.5	122	124.8
6 years	102.5	105	109	112	115.3	118.6	122	125	128
6.5 years	105	108	111.7	115	118.3	121.7	125	128.5	131.8
7 years	107.5	111	114.2	117.8	121.3	124.8	128	131.5	135
7.5 years	110	113	117.3	120.7	124.3	128	131.5	135	138.5
8 years	113	116.5	120	123.7	127.3	131	134.8	138	142
8.5 years	115	119	122.7	126.5	130.1	134	137.7	141.5	145
9 years	117	121	125	129	132.8	136.7	140.5	144.5	148.5
9.5 years	119.5	122.5	127.5	131.5	135.6	139.7	144	148	152
10 years	121.5	126	130	134	138.4	142.7	147	151	155.5
10.5 years	123.5	128	132.5	137	141.3	145.8	150	154.5	159
11 years	125.5	130	135	139.5	144.1	148.8	153.2	158	162.2
11.5 years	128	133	137.5	142	146.9	151.8	156.2	161	166
12 years	131	135	140	145	149.8	154.5	159	164	169
12.5 years	133.5	138.5	143	147.9	152.6	157.5	162	167	171.5
13 years	137	141.5	146	150.7	155.3	160	164.5	169	174
13.5 years	140	144	148.7	153	157.7	162	167	171	175.8
14 years	142	146.5	151	155	159.6	164	168.5	173	177
14.5 years	144	148.5	152.7	157	161.1	165.5	169.8	174	178
15 years	146	149	154	158	162.2	166.5	170.5	175	179
15.5 years	146.5	150.5	154.7	158.7	162.9	167	171	175	179.5
16 years	147	151	155	159	163.2	167.2	171.5	175.5	180
16.5 years	147.5	151	155.3	159.3	163.5	167.5	171.5	175.5	179.5
17 years	147.5	151	155.3	159.4	163.5	167.5	171.5	175.5	179.5
17.5 years	147.5	151	155.3	159.4	163.5	167.5	171.5	175.5	179.5
18 years	147.5	151	155.3	159.4	163.5	167.6	171.5	175.5	179.5

* Developed from UK-WHO growth charts. Growth charts may also be used to assign a score for step 3 of STAMP

© 2008 Central Manchester and Manchester Children's University Hospitals NHS Trust
Supported by an educational grant from Abbott.
Date Reviewed: July 2022 UK-N/A-2200464

Supplementary Material S2.

A modified screening tool for the assessment of malnutrition in pediatrics

Criteria	Screening Frequency			
	Week 1	Week 2	Week 3	Week 4
	Wt =	Wt =	Wt =	Wt =
	Ht =	Ht =	Ht =	Ht =

**Step 1**<br>Diagnosis (check the diagnosis and circle corresponding score)				
○ Bowel failure, intractable diarrhea<br>○ Burns and major trauma<br>○ Crohn's disease<br>○ Cystic fibrosis<br>○ Dysphagia<br>○ Liver disease<br>○ Major surgery<br>○ Multiple food allergies/intolerances<br>○ Oncology on active treatment<br>○ Renal disease/failure<br>○ Inborn errors of metabolism<br>○ **Respiratory Infection**<br>○ **Neonatal Disorders**	**3**	**3**	**3**	**3**
○ Behavioral eating problems<br>○ Cardiology<br>○ Cerebral palsy<br>○ Cleft lip and palate<br>○ Celiac disease<br>○ Diabetes<br>○ Gastro-esophageal reflux<br>○ Minor surgery<br>○ Neuromuscular conditions<br>○ Psychiatric disorders<br>○ Respiratory syncytial virus (RSV)<br>○ Single food allergy/intolerance<br>○ **Pneumonia**<br>○ **Acute diarrhea**	**2**	**2**	**2**	**2**
○ Day case surgery<br>○ Investigations	**0**	**0**	**0**	**0**

**Step 2**<br>Nutritional intake (What is the child's nutritional intake?)				
○ None (no nutritional intake)	3	3	3	3
○ Recently decreased/poor	2	2	2	2
○ No change/good	0	0	0	0

**Step 3**<br>Weight and height<br>**Use a Saudi growth chart or the centile quick reference tables to determine the child's measurements**				
○ > 3 centiles/columns apart (or weight < 2nd centile)	3	3	3	3
○ > 2 centiles/columns apart	1	1	1	1
○ Similar centiles/ columns	0	0	0	0

**Total scores**				
**Data**				
**Time**				
**Nurse's signature**				

Infant Weight and Height Centile Table (Girls)

Weight centile (kg)									
Age	3rd	5th	10th	25th	50th	75th	90th	95th	97th
Birth	2.5	2.6	2.7	2.9	3.1	3.5	3.7	3.8	4.1
2 months	3.7	3.9	4	4.3	4.8	5	5.5	5.7	6
4 months	4.9	5.1	5.4	5.8	6.3	7	7.5	7.9	8.3
6 months	5.7	5.9	6.1	6.7	7.3	8	8.6	9	9.3
8 months	6.4	6.6	6.9	7.5	8.2	8.8	9.7	10.1	10.4
10 months	6.9	7.1	7.5	8	8.7	9.7	10.5	11	11.4
12 months	7.2	7.5	7.9	8.5	9.4	10.2	11	11.6	12
14 months	7.5	7.7	8.2	8.9	9.7	10.6	11.5	12.1	12.5
16 months	7.8	8.1	8.5	9.25	10.1	11.1	12	12.6	13
18 months	8.1	8.4	8.7	9.5	10.5	11.5	12.5	13.1	13.5
20 months	8.4	8.6	9.1	9.9	10.9	11.9	12.95	13.6	14
22 months	8.6	8.9	9.4	10.2	11.2	12.25	13.4	14	14.5
24 months	8.8	9.1	9.6	10.5	11.5	12.6	13.7	14.5	15
Height centile (cm)									
Age	3rd	5th	10th	25th	50th	75th	90th	95th	97th
Birth	46	46.5	47	48.8	50	51.5	53	54	55
2 months	51.5	52.5	53	55	57	58	60	61	62
4 months	55.5	56	57	59	62	63.5	65	66	67
6 months	59.8	60.5	61.5	63	66	67.5	70.5	71.5	72
8 months	62.3	63	65	67	68.5	72	74	76	77
10 months	65	66	67	68	72	74	77	78.5	79
12 months	67	67.5	68	71	73	77	79	81	82
14 months	68.5	70	71	73	76	78.5	81.5	82.5	84
16 months	71	71.5	73	75.5	77.5	82	83	85	86
18 months	72	72.5	74	77	80	82	85.5	87.5	88
20 months	73	74	77	78.5	82.5	84.5	87.5	89	90
22 months	75	76	78	80	83	87.5	91	92.5	93
24 months	76	77	78.5	82	85	88	92	93	94

Source: Mohammad I. El Mouzan, Abdullah A. Al Salloum, Abdullah S. Al Herbish, Peter J. Foster, Mansour M. Qurashi, Ahmad A. Al Omar. The 2005 Growth Charts for Saudi Children and Adolescents (No. AR-20-63). King Abdulaziz City for Science and Technology 2009, Riyadh, KSA.

Infant Weight and Height Centile Table (Boys)

Weight centile (kg)									
Age	3rd	5th	10th	25th	50th	75th	90th	95th	97th
Birth	2.4	2.5	2.7	2.9	3.2	3.5	3.8	4	4.2
2 months	4	4.1	4.3	4.8	5.3	5.9	6.2	6.8	7
4 months	5.1	5.3	5.5	6	6.5	7.2	7.8	8.2	8.5
6 months	5.9	6.1	6.5	7.2	7.7	8.5	9.3	9.6	10
8 months	6.5	6.8	7.1	7.7	8.5	9.4	10.1	10.5	10.8
10 months	6.9	7.2	7.6	8.4	9.2	10	10.8	11.4	11.8
12 months	7.2	7.55	8	8.8	9.8	10.6	11.4	11.8	12.3
14 months	7.6	8	8.4	9.2	10.2	11.1	11.9	12.4	12.8
16 months	8	8.3	8.8	9.6	10.5	11.5	12.4	13	13.3
18 months	8.3	8.6	9.1	10	10.9	11.9	12.9	13.4	13.8
20 months	8.5	8.9	9.3	10.2	11.2	12.2	13.2	13.8	14.2
22 months	8.8	9.1	9.6	10.5	11.5	12.6	13.6	14.3	14.6
24 months	9	9.3	9.9	10.8	11.8	12.9	13.9	14.5	15
Height centile (cm)									
Age	3rd	5th	10th	25th	50th	75th	90th	95th	97th
Birth	46.5	47.5	48.5	50.5	51.3	52	54	55.5	61
2 months	52	52.5	53	55	57	58	61	62	62,5
4 months	57	57.5	58.5	60.5	62.5	65	67	68	68.5
6 months	60.5	61	62.5	64.5	67	69	71.5	72.5	73
8 months	63	64	65.5	67.5	70	72.5	75	76	77
10 months	65	66	67.5	70	72.5	75	77.5	78	79
12 months	67.5	68	70	72	75	77.5	80	81.5	82.5
14 months	68.5	70.5	72	74.5	77.5	80.5	82.5	84	85
16 months	71	72.5	73.5	77	79	82.5	85	86	87.5
18 months	73	74	75.5	78	81.5	83.5	87	88	89
20 months	74	75	76	79.5	82.5	85.5	88	90	92
22 months	76	77	78	81.5	84	87.5	90.5	92	93
24 months	77	78	80	83	86	89	90	93.5	95

Source: Mohammad I. El Mouzan, Abdullah A. Al Salloum, Abdullah S. Al Herbish, Peter J. Foster, Mansour M. Qurashi, Ahmad A. Al Omar. The 2005 Growth Charts for Saudi Children and Adolescents (No. AR-20-63). King Abdulaziz City for Science and Technology 2009, Riyadh, KSA.

Child Weight and Height Centile Table (Girls)

Weight centile (kg)

Age	3rd	5th	10th	25th	50th	75th	90th	95th	97th
Birth	2.5	2.6	2.7	2.9	3.1	3.5	3.7	3.8	4.1
2 years	8.8	9.1	9.6	10.5	11.5	12.6	13.7	14.5	15
2.5 years	9.5	9.8	10.4	11.3	12.4	13.6	14.8	15.6	16.3
3years	10	10.4	11	11.9	13.2	14.6	16	16.7	17.5
3.5 years	10.5	10.9	11.5	12.6	14	15.5	17	18	18.5
4 years	11	11.5	12.2	13.3	14.8	16.5	18.1	19.1	19.8
4.5 years	11.5	12	12.8	14.1	15.6	17.5	19.2	20.4	21.2
5 years	12.2	12.6	13.4	14.8	16.7	18.6	20.6	21.8	22.8
5.5 years	13	13.5	14	15	17	18	21	22.5	24
6 years	13.5	14	15	16.5	18.5	21	23	25	26
6.5 years	14	14.5	16	17.5	20	22.5	25	27	28
7 years	14.5	15.3	16.2	18.5	21	24	27	28.5	30
7.5 years	15	16	17	19.5	22	22.5	28.5	31	32.5
8 years	16	17	18	20.5	23.5	27	30.5	33	35
8.5 years	17	17.5	19	22.5	25	29	33	36	38
9 years	17.5	18.5	20	23	26.5	31	32.5	38	40
9.5 years	18.5	19.5	21	24	28	33	38	41	43.5
10 years	19.5	20.5	22	26	30	35.5	41	44.5	47
10.5 years	21	22	24	28	32.5	38	44	48	51
11 years	22	25	25.5	29.5	35	40.5	47	51.5	55
11.5 years	23.5	25	27	31.5	37	43.5	50.5	55.5	59
12 years	25	26	29	33	39	46	53.5	58.5	62
12.5 years	26.5	28	31	35.5	42	49	57	62	65.5
13 years	28	29.5	32	37	44	51.5	60	65	69
13.5 years	29.5	31	34	39	46	54	62	68	72
14 years	31	32.5	35.5	41	48	56	64	70	74

Height centile (cm)

Age	3rd	5th	10th	25th	50th	75th	90th	95th	97th
Birth	46	46.5	47	48.8	50	51.5	53	54	55
2 years	76	77	78.5	82	85	88	92	93	94
2.5 years	80	81	83	86.5	90	93	96	98	99
3years	83	84.5	86	90	93	97	100	102.5	103
3.5 years	87	88	90	93	97	100.5	103	105.5	106
4 years	90	91	93	96	100	103	107	108	110
4.5 years	93	94	96	98.5	103	107	110.5	112.5	113
5 years	96	97.5	99	102.5	107	110	113	116	117
5.5 years	99	101	102.5	106	110	113	117	119	120
6 years	103	104	106	109	113	117	121	122	123.5
6.5 years	105	106	108	112.5	115.5	120	123	125.5	127
7 years	106.5	109	111	115	119	122.5	126	129	130.5
7.5 years	110	112.5	114	117.5	122	126	129.5	132	133.5
8 years	112.5	113	116	120	123	127.5	132	134.5	135.5
8.5 years	114.5	116	118	122.5	126	131	135	136	137
9 years	116	118	121	125	129	133	137	139.5	141
9.5 years	120	121	123	127	132	136	140	142	143
10 years	122	123	126	130	134	138	142	145	146
10.5 years	124	126	128	132	137	141.5	145.5	148	150
11 years	126.5	129	132	135	140	145	149	152	153
11.5 years	130	132	134	138	143	147.5	152	155	156
12 years	132.5	134	137	141	145.5	150	154	157.5	159
12.5 years	135	136.5	139.5	142.5	148	152.5	157	159	161
13 years	137.5	139.5	142	145.5	150.5	155	159.5	161	162
13.5 years	140	141	144	147.5	152	156	160	162.5	164.5
14 years	141	142.5	145	149	153	157.5	161	163	165

Source: Mohammad I. El Mouzan, Abdullah A. Al Salloum, Abdullah S. AlHerbish, Peter J. Foster, Mansour M. Qurashi, Ahmad A. Al Omar. The 2005 Growth Charts for Saudi Children and Adolescents (No. AR-20-63). King Abdulaziz City for Science and Technology 2009, Riyadh, K

Child Weight and Height Centile Table (Boys)

Weight centile (kg)

Age	3rd	5th	10th	25th	50th	75th	90th	95th	97th
Birth	2.4	2.5	2.7	2.9	3.2	3.5	3.8	4	4.2
2 years	9	9.3	9.9	10.8	11.8	12.9	13.9	14.5	15
2.5 years	9.7	10.1	10.6	11.6	12.7	13.8	15.1	15.8	16.4
3years	10.5	10.8	11.4	12.4	13.6	15	16.3	17.1	17.6
3.5 years	11.1	11.5	12.1	13.2	14.5	16	17.5	18.4	19
4 years	11.6	12.1	12.7	13.9	15.4	16.9	18.6	19.5	20.2
4.5 years	12.2	12.6	13.3	14.6	16.2	17.9	19.6	20.7	21.5
5 years	12.6	13.2	13.8	15.3	17	18.8	20.8	22	22.8
5.5 years	13	14	14.5	16	18	20	22	23.5	24.5
6 years	14	14.5	15	17	19	21	23.5	25	26
6.5 years	14.5	15	16	17.5	20	22.5	25	26.5	28
7 years	15	15.5	16.5	18.5	21	24	26.5	28.5	29.5
7.5 years	15.5	16.5	17.5	19.5	22	25	28	30.5	32
8 years	16	17	18	21	23.5	27	30	33	34
8.5 years	17	18	19	22	25	29	32.5	35	37
9 years	17.8	18.8	20	23	26.5	31	35	38	39.3
9.5 years	18.4	19.4	21	24	28	33	37.5	40.5	43
10 years	19	20.4	22	25.4	30	35	40	44	46
10.5 years	20	21	23	27	31.5	37	43	47	49.5
11 years	21	22	24	28	33	39.5	46	50	53
11.5 years	22	23	25.5	29.5	35	42	49	54	57
12 years	23	24.5	27	31.5	37.5	45	52	57.5	61
12.5 years	24.5	26	28	33	39.5	47.5	55.5	61	65
13 years	25.5	27.5	30	34	42	50	59	65	69
13.5 years	27.5	29	31.5	37	44	53	62	68.5	73
14 years	29	30.5	33.5	39	47	56	65	72	76.5

Weight centile (kg)

Age	3rd	5th	10th	25th	50th	75th	90th	95th	97th
Birth	46.5	47	47.5	48.5	51.3	52	54	55.5	61
2 years	77	78	80	83	86	89	90	93.5	95
2.5 years	81	82.5	84	87	90	93.5	97	98.5	100
3years	84.5	86	88	91	94	97.5	101	103	104
3.5 years	88	89	91.5	94	98	102	105	106	107
4 years	91	92.5	94.5	98	102	105.5	108	111	112
4.5 years	94	96	97.5	101	105	108	112	113.5	115.5
5 years	97	98	101	104.5	107	112	115	117	119
5.5 years	100	101	103	107	110	115	118	120	122
6 years	102.4	103	106	109.5	113	116	121	123	125
6.5 years	105	107	108	112.5	116	120	124	126	127.5
7 years	107.5	109	111	115	118	123	126	128	130
7.5 years	110	112	114	118	122	126	130	132	133
8 years	133	114	116	120	123	127	133	134	136
8.5 years	116	117	119	123	127	131	135	137	139
9 years	118	119	122	125	129	133	137	140	142
9.5 years	120	122	124	127	132	136	140	143.5	145
10 years	122	123	126	129	134	138	142.5	145	147
10.5 years	124	125.5	127.5	132	136	140	145	147.5	150
11 years	126	127.5	130	133.5	137.5	143	147	151	152.5
11.5 years	128	130	132	136	141	146	151	154	155
12 years	131	132	135	138	144	149	153	157	159
12.5 years	134	135	137	142	147	152	157	160	162
13 years	135	137	140	145	150	155	160	163	165
13.5 years	137.5	138	142.5	157.5	152.5	157.5	162.5	166	168
14 years	140	142.5	145	150	155	161	166	169	171

Source: Mohammad I. El Mouzan, Abdullah A. Al Salloum, Abdullah S. AlHerbish, Peter J. Foster, Mansour M. Qurashi, Ahmad A. Al Omar. The 2005 Growth Charts for Saudi Children and Adolescents (No. AR-20-63). King Abdulaziz City for Science and Technology 2009, Riyadh, KSA.

Supplementary Material S3.

Nutrition Status of the Patients Based on Anthropometric Measurements

Variables	
Weight-for-height Z-score (WHO/CDC) * (age <59 months)	N=155
Severe malnutrition	16 (10.3%)
Moderate malnutrition	7 (4.5%)
Mild malnutrition	25 (16.1%)
Normal	65 (41.9%)
Overweight	16 (10.3%)
Obese	14 (9%)
Very obese	12 (7.7%)
Weight-for-height Z-score (Saudi)* (age <59 months)	N=155
Severe malnutrition	11 (7.15%)
Moderate malnutrition	5 (3.2%)
Mild malnutrition	20 (12.9%)
Normal	47 (30.3%)
Overweight	38 (24.5%)
Obese	23 (14.8%)
Very obese	11 (7.15%)
BMI-for-age Z-score (CDC) (age > 59 months) *	N=152
Severe malnutrition	15 (9.9%)
Moderate malnutrition	16 (10.5%)
Mild malnutrition	12 (7.9%)
Normal	73 (48.0%)
Overweight	22 (14.5%)
Obese	12 (7.9%)
Very obese	2 (1.3%)

BMI-for-age Z-score (Saudi) (age > 59 months) *	N=152
Severe malnutrition	8 (5.3%)
Moderate malnutrition	8 (5.3%)
Mild malnutrition	23 (15.1%)
Normal	36 (23.7%)
Overweight	45 (29.6%)
Obese	24 (15.7%)
Very obese	8 (5.3%)
Weight-for-height Z-score (WHO/CDC) *	N=155
Malnutrition	48 (31%)
Absence of malnutrition	107 (69%)
Weight-for-height Z-score (Saudi) *	N=155
Malnutrition	36 (23.2%)
Absence of malnutrition	119 (76.8%)
BMI-for-age Z-score (CDC) *	N=152
Malnutrition	43 (28.3%)
Absence of malnutrition	109 (71.7%)
BMI-for-age Z-score (Saudi) *	N=152
Malnutrition	39 (25.7%)
Absence of malnutrition	113 (74.3%)
